# Recent Advances on the Function and Purification of Milk Exosomes: A Review

**DOI:** 10.3389/fnut.2022.871346

**Published:** 2022-06-09

**Authors:** Xiaoping Li, Lan Su, Xinling Zhang, Qi Chen, Ying Wang, Zhenwei Shen, Tian Zhong, Ling Wang, Ying Xiao, Xiao Feng, Xi Yu

**Affiliations:** ^1^Faculty of Medicine, Macau University of Science and Technology, Taipa, Macao SAR, China; ^2^Shulan International Medical College, Zhejiang Shuren University, Hangzhou, China; ^3^Guangdong-Hong Kong-Macau Joint Laboratory for Contaminants Exposure and Health, Guangzhou, China; ^4^College of Food Science and Engineering/Collaborative Innovation Center for Modern Grain Circulation and Safety/Key Laboratory of Grains and Oils Quality Control and Processing, Nanjing University of Finance and Economics, Nanjing, China

**Keywords:** exosomes, purification technique, biological function, clinical application, milk

## Abstract

Exosomes are nano-scale extracellular vesicles, which can be used as drug carriers, tumor treatment, intestinal development and immune regulator. That is why it has great potential in pharmacy, functional foods, nutritional supplements, especially those for infants, postoperative patients, chemotherapy patients and the elderly. In addition, abnormal exosome level is also related to diseases such as cardiovascular diseases, tumor, diabetes, neurodegenerative and autoimmune diseases, as well as infectious diseases. Despite its high biological significance, pharmaceutical and nutritional value, the low abundancy of exosomes in milk is one of the bottlenecks restricting its in-depth research and real-life application. At present, there is no unified standard for the extraction of breast milk exosomes. Therefore, choosing the proper extraction method is very critical for its subsequent research and development. Based on this, this paper reviewed the purification techniques, the function and the possible applications of milk exosomes based on 47 latest references. Humble advices on future directions, prospects on new ideas and methods which are useful for the study of exosomes are proposed at the end of the paper as well.

## Introduction

**FIGURE 1:**
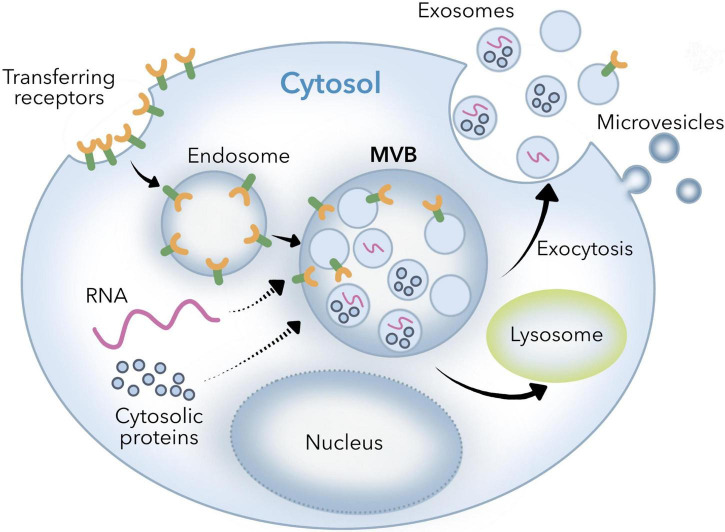
Biogenic mechanism of exosomes.

Cells produce small vesicles through endocytosis, and small vesicles fuse to form early endosomes. Under the coordinated regulation of cytosolic proteins, early endosomes sprout to form multivesicular bodies containing multiple intracavity small vesicles, which fuse with the plasma membrane of the cell and release to the outside of the cell through exocytosis, called exosomes (1). This is the biogenesis and secretion process of exosomes (Figure 1), these exosomes are a type of extracellular vesicles, which contain lipids, proteins, miRNA, and other contents (2). Exosomes, 30–150 nm nanostructures secreted from donor cells and internalized by recipient cells, can play an important role in the cellular entry of some viruses, these exosomes are actively secreted into various body fluids, including blood, urine, saliva, cerebrospinal fluid, and human breast milk (HBM) (3). In 1983, exosomes were first discovered in the reticulocytes (immature red blood cells) of mature mammals (4). With the deepening of research, it has been discovered that exosomes play an important role in the exchange of information between cells (5, 6), and they can be utilized as drug carriers and biomarkers for disease diagnosis, etc. (7). For example, in neurodegenerative diseases, aggregates of β-amyloid bind? circulating exosomes have been shown to reflect the neuropathic progression of patients. By detecting levels of circulating exosomes positive for β-amyloid, such change can be observed (8, 9). Exosomes can also be involved in autoimmune diseases through the production of immune complexes, antigen presentation, and coagulation. Exosomes released by virus-infected cells can deliver viral RNA to dendritic cells and macrophages, thereby activating pattern recognition receptors of recipient cells, inducing the expression of type I interferon and pro-inflammatory cytokines, and enhancing antiviral and antibacterial abilities of the body (10, 11). Exosomes have been isolated from different body fluids such as blood, urine, saliva, milk, amniotic fluid, and ascites (3). Milk is an important food source for the mammals and can be obtained in large quantities from the mammals under the lactation period. It is safe and can be obtained from a wide range of sources at a relatively low cost (12). In-depth study of exosomes showed that exosomes and their RNA can be generated not only by endogenous synthesis but also from dietary sources. For example, bovine milk exosomes and their RNA can be transferred between human and bovine species (13). In addition, there are a large number of bioactive substances in breast milk exosomes, including growth factors, cytokines, antibodies, lactoferrin, oligosaccharides, and microRNAs (miRNA) (14–17), which help mammal infants resist infection and inflammation, promote the maturation of immune system and gastrointestinal function, and promote growth and development (18–21). Therefore, it is challenging to mimic such ingredients in formula milk (22). That is the reason researchers began to investigate the possibility of using the exosomes from cow, sheep and even humans to enhance the formula milk (23, 24).

Given the physiological functions of breast milk exosomes, it is of great significance to study how to isolate and purify breast milk exosomes. Through the isolation and purification of human milk exosomes, these valuable bioactive exosomes can be better studied and utilized (25, 26). At present, the extraction methods of breast milk exosomes can be generally divided into ultracentrifugation, membrane ultrafiltration, density gradient centrifugation, polymer precipitation method, size exclusion method (27), immunocapture method (28), and microfluidic technology (29, 30), and each of these technologies has its advantages and disadvantages (Table 1). Density gradient centrifugation has high purity, but the centrifugation time is difficult to control. Immunocapture method based on antigen-antibody specific binding can selectively isolate breast milk exosomes, but the ungentle elution conditions will cause inactivation of breast milk exosomes, and it is not suitable to generate a large number of samples (31). In practical experimental operations and clinical studies, the most suitable breast milk exosomes extraction method is usually selected according to factors such as sample type, downstream experiment, research target, and hard experimental conditions. Ultracentrifugation is still the gold standard method for extracting breast milk exosomes, but it still has a series of shortcomings and problems, owing to the small size of breast milk exosomes. Furthermore, there are many characteristics of outer breast milk exosomes surface, which has a lot of specificity of protein expression, such as an antigen, heat shock protein, the major histocompatibility complex, etc. Surface selectivity can even be used to screen breast milk exosomes specifically. In addition, density gradient centrifugation combined with ultracentrifugation can also achieve high purity extraction of breast milk exosomes with a density between 1.13 and 1.19 g/mL, and avoid the disadvantage of the aggregation of breast milk exosomes at the bottom (32). In recent years, the demand for breast milk exosomes in biological research and clinical application is increasing day by day, and the corresponding breast milk exosomes extraction technology is emerging in an endless stream. Professionals from biology, materials science, physics, and other fields give full play to their advantages and innovation ability to promote the development of breast milk exosomes extraction technology, and accelerate the research and clinical application of breast milk exosomes.

**TABLE 1:** Comparison of the current breast milk exosomes extraction methods.

Method	Time (h)	Instrumental requirement	Consumable requirement	Recovery rate	References
Ultracentrifugation	3	Ultracentrifuge	No	5-25%	(26, 37, 39, 45, 38)
Density gradient centrifugation	3	Ultracentrifuge	Sucrose	60%	(23, 24, 49, 51, 52)
Polymer precipitation	2	Chromatograph	Polyethylene glycol and Chromatographic column	50%	(53–56)
Size exclusion method	3	Chromatograph	Chromatographic column	70%	(21, 27, 62, 63)
Immunocapture method	1.5	No	magnetic beads	70%	(28)
Microfluidic technology	1	Microfluidic devices	microfluidic	80%	(50)

Human breast milk contains protein, lipids, lactose, minerals, vitamins, and other substances, and is rich in lipids. Lipids (mainly triacylglycerols) are released into milk fat globules (MFG) through breast epithelial cells. These MFGs are lipid droplets surrounded by three layers of complex phospholipids of proteins and glycoproteins. Due to the similar structure of MFG and exosomes, MFG is the main interference during the extraction of exosomes (33, 34).

**FIGURE 2:**
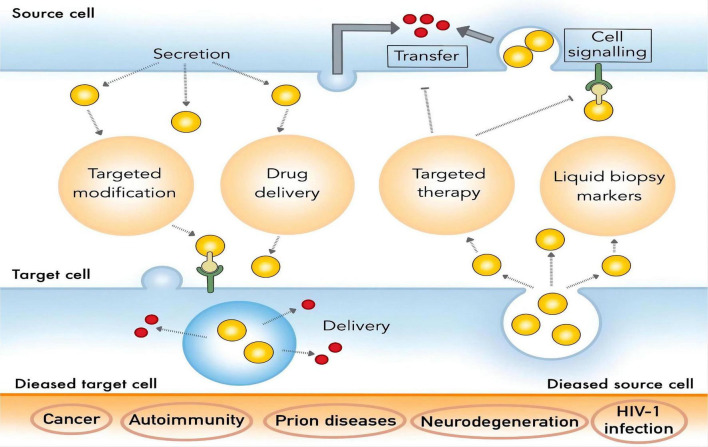
Clinical application of HBM exosomes.

With the in-depth study of the physiological function and mechanism of breast milk exosomes, HBM exosomes are expected to play an important role in the research and development of functional foods such as infant formula milk and functional drinks as new prebiotics. At present, researchers do not have a unified standard for the extraction of HBM exosomes, and existing extraction methods have different pros and cons. Although HBM exosomes show exciting application prospects in disease diagnosis, drug delivery, and nutritional supplementation, there are still lots of unsolved issues about them. For example, firstly, exosomes have problems such as high cost of extraction and complicated operation during the research process. Secondly, the test samples contain many components similar to exosomes, such as cell debris and other extracellular vesicles or microvesicles, which will interfere with the accuracy of identification. Finally, the limited drug loading capacity of exosomes may not meet the therapeutic needs. Among these, the extraction techniques are crucial for studying and making use of these valuable exosomes (35). This article reviews the potential for extraction of HBM exosomes and their clinical application (Figure 2), which will lay the foundation for future research and development of HBM exosomes.

## Extraction Method of Breast Milk Exosomes

Breast milk contains a large amount of biological macromolecules, which interferes with the detection and analysis of exosomes. The complexity of human samples and the heterogeneity of exosomes increase the difficulty to extract exosomes from HBM. The size and concentration of purified HBM exosomes were measured by nanoparticle tracking, while western blotting was used to confirm the presence of the exosome-related proteins CD9 and CD63, lactoferrin, T cell immunoglobulin and secretory immunoglobulin A (SIgA). It is urgent to explore the application of efficient separation and enrichment of exosomes from complex HBM in clinical practice.

### Ultracentrifugation

Ultracentrifugation is currently the most frequently used and reported method for extracting breast milk exosomes and is considered the “gold standard” for exosomes extraction (36). The ultracentrifugation method is based on the difference in the sedimentation speed of exosomes, proteins, cells, and other substances in the sample. Through different centrifugal forces and time, the supernatant and sediment are separated. The separation efficiency of exosomes depends not only on the centrifugal force but also on the type of rotor, κ-factor, and solution viscosity (37). Compared with the horizontal rotor, the fixed-angle rotor has a shorter sedimentation path length and have a better separation effect. However, the disadvantage of this method is that the obtained breast milk exosomes are deposited on the tube wall instead of the bottom, which makes them quite mobile and compromises the yield. The κ-factor can measure the ball-forming efficiency of the rotor. The specific rotor parameters (t-time, in hours, κ-κ factor, s-sedimentation coefficient) need to be obtained to estimate the time required for the sedimentation of breast milk exosomes. The centrifugal conditions used in different laboratories can be compared and standardized (38). Viscosity is also an important parameter of breast milk exosomes. When using ultracentrifugation to process biological liquids, it is necessary to consider the impact of sample viscosity on the sedimentation rate and the size of breast milk exosomes in advance (39).

The ultracentrifugation method is easy to operate and can be followed by entry-level technicians. It is the most common method for breast milk exosome purification, requiring only one time of ultracentrifugation. Besides, the cost of ultracentrifuge is low and the consumption of solvents is minimal. It has gradually become the most popular choice for researchers. The basic idea of using ultracentrifugation to extract breast milk exosomes is as follows. First, the fat globules in the emulsion are removed by preliminary centrifugation, and then casein and cell debris in the emulsion are removed by second centrifugation. After that, the 0.22 μm filter can be used for pre-treatment of the sample to prevent the clogging of the delicate ultrafilter, and the sample is further filtered using proper ultrafilter with certain cut-off molecular weight (MW) to remove apoptotic bodies and small vesicle aggregates (40–42). Centrifugation is normally implemented to accelerate the separation processes, and to dissolve breast milk exosomes in PBS solution. According to experimental conditions, you can choose to further obtain breast milk exosomes with specific MW by ultracentrifugation. Sow milk exosomes were extracted by 110000 g centrifugation with SW41T rotor for 2 h, and it was found that sow milk exosomes were rich in miRNA promoting intestinal epithelial cell proliferation (43, 44). Izumi et al. (37) carried out ultracentrifugation at 100,000 *g*, 90 min, and washed with PBS to obtain milk exosomes. Later experiments showed that the miRNA in milk exosomes can be used by human macrophages ingest.

Although ultracentrifugation is a widely used method for extracting milk exosomes, it also has shortcomings. The preliminary treatment can remove some contaminants with relatively large physical size, but there are still other impurities in the sample with similar MW to milk exosomes. To further study the breast milk exosomes, especially in the field of proteomics, co-precipitation of protein aggregates in milk requires additional purification steps for qualitative and quantitative mass spectrometry analysis (7). In addition, ultracentrifugation is relatively time-consuming and may cause damage to the breast milk exosomes under high-speed centrifugation conditions, which will have a certain impact on downstream analysis (45).

### Density Gradient Centrifugation

Density gradient centrifugation is an improvement as compared to the traditional differential centrifugation. *Via* the introduce of an inert medium into the centrifugation system, the former transfers the exosomes to the corresponding density gradient range by ultracentrifugation for separation. Density gradient centrifugation is further divided into rate zone centrifugation and isopycnic gradient centrifugation. The former requires a precise density gradient centrifugation system (such as iodixanol) before separation, while the latter does not need to be pre-set, but requires a suitable “self-partitioning” centrifugation media (such as cesium chloride). After ultracentrifugation, exosomes can be dissolved and stored in a stable form in their corresponding density gradient range. Density gradient centrifugation does not require repeated centrifugation and can separate various substances in the sample within one time, and the physicochemical properties of exosomes are not affected due to the protection of the inert medium. Therefore, this method can be used to isolate exosomes with excellent integrity, while multiple types of substances in the sample can be simultaneously isolated for comparative studies. For example, Jeppesen et al. used density gradient centrifugation to simultaneously separate small vesicles (including exosomes) and non-vesicle fractions in cell culture fluids for comparative analysis (46). At present, OptiPrep™ iodixanol is normally used as a centrifugation medium for density gradient separation of exosomes, and the isolated exosomes showed high purity and moderate yield (47). In addition, the process of rate zone centrifugation is dynamic as the density of solutes including breast milk exosomes is greater than the density of the gradient medium. To prevent breast milk exosomes from precipitating, high-density spacers are often added to the bottom of the centrifuge tube (36). However, appropriate centrifugation time is also important to avoid impurities with similar densities.

As a special form of ultracentrifugation, density gradient centrifugation can remove protein aggregates with different densities and obtain high-purity breast milk exosomes. The disadvantage is that the operation is complicated and time-consuming. The principle underlying the density gradient method is similar to that of ultracentrifugation, except that the crude exosomes obtained from density gradient centrifugation need to be placed in a density gradient medium and centrifuged for further purification. The density of the HBM exosomes layer is 1.13–1.19 g/mL, which is their average density within the density gradient (48). This layer is thus carefully aspirated and redissolved in PBS. Currently, proteomics studies of breast milk exosomes tend to use the sucrose density gradient method (24, 49–52). The more classic method is the extraction method of Reinhardt when he studied the proteomics of bovine milk exosomes in 2012 (51). In this method, the purified milk exosomes were obtained in 43%/35% layer after centrifugation under four sucrose density gradients of 80, 43, 35, and 5%. In this way, the separation of milk exosomes from non-vesicle particles was successfully achieved. Therefore, this method separates vesicles from particles of different densities and can extract HBM exosomes existing in low content. This method has high purity, and the buffer effect of the separation medium can also reduce the damage to HBM exosomes by centrifugal force, which is suitable for functional research, marker detection, and content analysis of exosomes. Exosomes have been successfully isolated from cell culture supernatant and HBM using this method. However, this method is more complicated to operate, which limits its separation efficiency and its application in clinical and diagnostic research.

### Polymer Precipitation Method

The polymer precipitation method, also known as the chemical precipitation method, is originally used in virus research. The mechanism of this method is to combine water molecules by introducing drainage polymers, such as polyethylene glycol (PEG) (53), to change the dispersion status of the sample system so that components with low solubility can precipitate and thus be separated (54). By adding proper precipitant to the supernatant, breast milk exosomes precipitation can be obtained by centrifugation. Zhou et al. (55) extracted HBM exosomes with ExoQuick Precipitation Kit and showed that it was rich in immune-related miRNA. Lukasik et al. (56) extracted HBM exosomes and sow milk exosomes using precipitation kits and analyzed their miRNA, it is indicating that mammalian milk exosomes are rich in miRNA. Rani et al. (57) used the same precipitation kits to extract exosomes from HBM. By establishing a new *in vitro* digestion model and doing cell experiment, it was shown that HBM exosomes protect miRNA from severe digestion processes. Thus, HBM exosomes can cross the intestinal barrier into blood circulation and obtain cell function.

Both chemical precipitation and ultracentrifugation are non-specific methods for separating breast milk exosomes. That is why its pretreatment and post purification process is important. Subcellular particles such as lipoprotein can be removed in the pre-separation step, and the polymer can be removed by the Sephadex G-25 column in the post-separation step. Aside from the other components which are hard to remove from the sample, the precipitation reagent itself also brings in interferences, such as PEG, which can cause severe noise during proteomic analysis. PEG is an ion inhibitor and can lead to + 44 ion series in the mass spectrum (58). As a non-volatile solute, PEG can prevent matrix crystallization, but will pollute the liquid chromatographic column of matrix-assisted laser desorption ionization time of flight mass spectrometry (MALDI-TOF-MS) (59). However, such a dilemma can be solved by choosing proper sample pre-treatment methods such as gel digestion or proteomics research (60, 61). Other unsolved issues in the precipitation method using PEG include low purity and recovery, miscellaneous protein impurities (false positive), uneven particle size, polymer contaminants, and spoilage of HBM exosomes caused by chemical additives such as Tween-20, etc. These unsolved issues will affect the biological activity of HBM exosomes to a certain extent.

### Size Exclusion Method

The size exclusion is a method of extracting breast milk exosomes according to the size of exosomes. At present, the extraction of milk exosomes by the size exclusion method is mostly combined with ultracentrifugation. Milk fat globules, cell fragments, and other substances are removed in the early stage. Exosomes are then obtained by ultra-centrifugation, which is then eluted and purified by a chromatographic column (61, 62). Blans et al. (27) successfully isolated extracellular vesicles from human and bovine milk by the size exclusion method, and found that the expression of marker proteins such as CD9, CD63, and CD81 monoclonal antibodies were higher by the size exclusion method without ultracentrifugation. Vaswani et al. extracted breast milk exosomes by ultracentrifugation combined with the size exclusion method and iodixanol density gradient method (63, 64). The results showed that ultracentrifugation combined with the size exclusion method had higher HBM exosomes yield.

The main advantages of size exclusion method are simple operation, high enrichment efficiency, good reproducibility, and high sample purity. In addition, the size exclusion method relies on gravity flow and low pressure to separate breast milk exosomes, so it can obtain unruptured breast milk exosomes with regular shape. However, the chromatographic column limits the sample loading amount, generally no more than 0.5 mL, which reduces the extraction efficiency of breast milk exosomes. That is why the method is time-consuming and is not suitable for batch processing. This method is only suitable for functional study, marker detection and content analysis of breast milk exosomes. In addition, multiple chromatographic columns are normally used due to the existence of protein and lipid interference in these exosomes, which limits the application of this method (65).

### Immunocapture Method

Proteomics research of milk exosomes revealed that aside from common proteins derived from different cells, breast milk exosomes also contain cell-specific proteins, which can be used as markers for their immune separation. The surface of HBM exosomes contains many targeting markers, such as CD9, CD63, and CD81 (27, 54), or annexin from the four-transmembrane protein family (66), which can be selectively combined with specific antibodies for their extraction. At present, immunocapture methods are available in the form of various kits. The most commonly used immunocapture kits are Enzyme-linked immunosorbent assay (ELISA), and immunomagnetic beads are becoming popular in recent years (28). ELISA uses purified antibodies, such as CD9 antibody, to coat a microplate, and sample incubation is carried out using the microplate to extract breast milk exosomes. The immunomagnetic bead method uses CD9, CD63, and other antibodies to modify the surface of the magnetic beads, and sample incubation with the addition of these magnetic beads is conducted to adsorb HBM exosomes with the corresponding surface ligand *via* antibody-antigen interaction. Compared with the ELISA microplate, the magnetic beads have a larger contact surface area and is easy to disperse in large size sample, and the obtained exosomes tend to stay in their intact structure due to the rapid magnetic separation (67).

The most representative extraction method in immunocapture is magnetic bead extraction, and the main advantage is that magnetic beads provide a large surface area for exosome capture to improve the extraction efficiency. Because magnetic bead extraction can separate exosomes from specific cells, and this method is highly specific, the purity of the product is high and is suitable for marker detection and clinical diagnosis of exosomes (68, 69). However, its application is limited to samples with a certain surface target for immune adsorption and the cost is high. In addition, the ELISA method and the immunomagnetic bead method require multiple times of washing during the extraction process, which contributes to the loss of exosomes (70). At present, there are few reports on the extraction of breast milk exosomes by the immunocapture method. Considering the complexity of the components of HBM, higher requirements are put forward for the pretreatment and purification of HBM. At the same time, the immunocapture method is not suitable for the processing of a large number of samples. Therefore, it is necessary to consider pre-processing of HBM and then concentrating it to achieve better results. In addition, this method has some problems, such as harsh use and storage conditions.

### Microfluidic Technology

Microfluidic technology is a recently developed technique for high demanding separation tasks. The microfluidic chips are designed for small-volume liquid samples and can act as lab-on-a-chip to achieve separation and reaction in their dedicated designed channels within a short time, which can be applied to exosome separation and downstream analysis. Microfluidic technology is a promising direction of liquid biopsy for its high efficiency and easy operability. Exosomes are targeted by the binding with specific antibodies immobilized on the inner capture surface of microfluidics. By making surface modifications on the inner walls of these tunnels, the separation of exosomes using microfluidics can be achieved based on different chromatographic principles. At present, microfluidic technology is mainly divided into three categories, which are separation based on size, separation based on immunoaffinity, and dynamic separation (29, 71).

Size-based milk exosomes separation devices usually include nano filters, nanoporous membranes, or nanoarrays, which are usually placed in cross-sections or fabricated on the substrate of a microfluidic channel. When the sample fluid flows through the channel, these exosomes will be trapped in the structures to achieve the separation effect. The principle of the extraction method of milk exosomes based on immunoaffinity is similar to the separation by immunocapture. By modifying the surface of the microchannel with antibodies or affinity particles or immuno-magnetic beads, the microfluidic device with immunoaffinity is used to collect these milk exosomes. According to specific biomarkers on the surface of milk exosomes, these exosomes containing the target protein are specifically separated from other extracellular vesicles (50). Considering the limitations and shortcomings of extraction methods based on size and immunoaffinity, some studies have combined or integrated the use of external forces such as electric field, acoustic force, and hydrodynamic force into the microfluidic design for faster and easier extraction of milk exosomes. These methods can be classified as dynamic separation methods. Zhao et al. (72) used a dialysis membrane of 30 nm pore size to capture exosomes on the membrane surface, while allowing proteins to flow through the membrane. Specifically, exosomes are manipulated by applying an electric field to a porous dialysis membrane (72). In this device, non-target nanoparticles were driven away, and exosomes were captured on the membrane concurrently by electrical forces. This method showed 65% exosomes recovery and 83.6% protein removal in a relatively short time (≈30 min). However, this method requires optimized electrical conditions for different liquid samples. Furthermore, the device has a complicated structure that need to be further simplified. Meanwhile, Lee et al. proposed a continuous and contactless “acoustic nanofilter” system for extraction of exosomes based on ultrasonic standing waves, in which these exosomes are separated by size and density through applying different acoustic forces (73). In this device, an acoustic field is applied across the sample flow field, forming pressure nodes at either side of the channel. Larger particles experience a much stronger acoustic force that moves more rapidly across the channel to the pressure nodes, and are removed by “sheath flows” within these regions. Because the filtration operates in a continuous-flow mode, the risk of channel clogging is minimized. This method has achieved a 80% recovery for exosomes and a 90% recovery for larger microvesicles from the cell culture medium. One remarkable advantage of this device is that particles of different sizes can be separated by tuning the magnitude and frequency of the acoustic field, as well as the flow rate. Complicated nanolithography is avoided in the fabrication of this device, although electrical and acoustic transducers are essential components. Furthermore, relevant technical acoustics knowledge is required for the proper operation of the device. Using another strategy, Yang et al. (30) applied flow field-flow fractionation (FlFFF) technology for the separation of subcellular species, including exosomes. In this case, the external force employed is a hydrodynamic force, and separation by FlFFF is achieved within a rectangular microchannel. Sample components of cell homogenates are carried by migration flow along the channel axis to the detector, with a separate flow moving across the channel, which results in extraction between sample components along one wall of the channel. Smaller particles diffuse faster and move further than larger particles against the channel wall in a direction opposite to the crossflow and smaller particles ultimately migrate faster down the channel, since the flow velocity of the parabolic flow profile increases as the distance from the channel wall increases.

The major limitation of microfluidics is the sample size and the recovery rate. Although size-based microfluidics can produce homogeneous exosomes that are not contaminated by non-exosome proteins and other extracellular vesicles, the recovery rate is low. The microfluidic separation method based on immunoaffinity achieves high exosome purity but can only isolate milk exosomes rich in target proteins. The dynamic extraction method can achieve the uniformity of milk exosomes size after extraction. However, it requires expensive equipment, such as microfluidic devices, and professional technicians (71). Besides, microfluidic technology is difficult to be applied on a large scale due to the lack of standardization, large-scale clinical sample testing, and relevant method validation (72). Ultimately, the extraction of breast milk exosomes based on microfluidic technology has great potential for the development of clinical devices that can be used in hospitals for diagnostics, as well as portable personal pre-diagnostic devices used in various samples, such as HBM and bovine breast milk (74).

## The Function and Application of HBM Exosomes

### Effects on Gastrointestinal Development Function in Infants

HBM provides essential nutrients for newborn growth and development and contains a variety of bioactive ingredients that can affect gastrointestinal tract development in breastfed infants. HBM also contains mRNA, microRNA, and lncRNA, most of which are encapsulated in human breast milk-derived exosomes and have an impact on important development-related biological functions (75). Exosomes found in preterm colostrum (PC) and term colostrum (TC) promoted VEGF protein expression, induced the proliferation and migration of small intestinal epithelial cells (FHCs) and induced angiogenic responses in endothelial cells (76). Exosomal circRNAs found in human colostrum (HC) may regulate VEGF signaling, and intestinal development (77, 78). HBM exosomes can influence gastrointestinal protection, and are important in the prevention and treatment of gastrointestinal diseases, which can regulate the metabolism of biological substances and promote the growth and development of infants (55, 79–83). HBM exosomes exert beneficial effects in preventing necrotizing enterocolitis (NEC) by reducing inflammation and injury to the intestinal epithelium as well as by restoring intestinal tight-junction proteins. HBM exosomes can promote intestinal maturation and microbiome establishment, which act as mediators for probiotics to function. HBM exosomes may cooperate with probiotics to regulate the intestinal microbiota of infants (18, 84–92), so as to improve the resistance to intestinal pathogens (Table 2).

**TABLE 2:** Beneficial effects of some human breast milk-isolated probiotic strains.

Bacterial species	Prebiotic effect	References
*L.salivarius* CECT5713	Intestinal engraftment; Produce antibacterial compounds; No D-lactic acid products Antibacterial activity Immune regulation Anti-inflammatory effects	(87) (89) (22) (90)
*L.gasseri* CECT5714	Intestinal engraftment Improved intestinal function Produce antibacterial compounds Antibacterial activity Immune regulation Antiallergic effect	(19) (19, 85) (91) (89) (86) (20)
*L. fermentum* CECT5716	Intestinal engraftment Produce antibacterial compounds Antibacterial activity Immune regulation Immune enhancement Anti-inflammatory effects	(18) (91) (89) (22) (18) (90)

### Effects on Immune System Function in Infants

HBM exosomes also have immune function (93), and these exosomes are known to regulate B cell, T cell, monocyte development, and to control the innate immune response and cytokine production. HBM exosomes contribute to physiological and therapeutic functions. Several studies have reported that HBM is rich in immune-regulating miRNA, such as let-7a, miR-21, miR-141, miR-146a, and miR-148a. Further research shows that exosomal miRNA may transfer genetic material to the infant, which can be protected from digestion in the baby’s gastrointestinal tract and circulate into the blood (Table 3). The Gabrielson group demonstrated that human breast milk-derived exosomes inhibited CD3-induced production of IL-2, IFN-γ, and TNF-α in peripheral blood mononuclear cells (PBMCs) while increasing the number of FoxP3 + CD4 + CD25 + regulatory T cells in PBMCs. They also reported that MUC-1 expressed on human breast milk-derived exosomes binded to DC-SIGN on monocyte-derived dendritic cells (MDDCs) and blocked viral transfer from MDDCs to CD4 + T cells *via* clinical research (94). In addition, HBM exosomes possess different phenotypes, depending upon maternal sensitization and lifestyle, and different phenotypes influence allergy development in children differentially (95). For example, exosomes derived from mothers with an anthroposophic lifestyle are associated with a lower prevalence of allergic sensitization than those from non-anthroposophic mothers. These data suggest that HBM exosomes potentially influence the immune system. The results revealed that exosomes from colostrum are highly enriched in proteins implicated in the innate immune response, inflammatory response, acute-phase response, platelet activation, cell growth, and complement activation, whereas proteins implicated in transport and apoptosis are enriched in exosomes from mature milk (96). Proteomic studies support the immunomodulatory function of HBM exosomes. Antigen peptide MHC complexes are presented to T cells through direct presentation or cross presentation to mediate their immune response to antigens, which indicate the importance of colostrum in immune defense and immune system development in infancy.

**TABLE 3:** Expression and functions of immune-related miRNA in human breast milk.

miRNA	Immune Function	References
miR-17 and miR-92 cluster	B-cell, T-cell, and monocyte development	(14)
miR-21	Inflammatory reaction	(82)
miR-29a	Suppression of immune responses to intracellular pathogens by targeting IFN-γ	(55)
miR-30b	Promotion of cellular invasion by directly targeting GalNAc transferase, immunosuppression by increasing IL-10; Tumor cell invasion and immunosuppression	(79, 55)
miR-106 miR-125b	Regulation of IL-10 production Negative regulation of TNF-α production, activation, and sensitivity Proliferation and differentiation of stem cells	(83) (14)
miR-146a	Regulation of TLR signaling pathway expression	(15, 17)
miR-146b	Negative regulation of the innate immune response by targeting NF-κB signaling, control of TLR and cytokine signaling; Dendritic cell apoptosis	(14, 15)
miR148a	Regulate Th1/Th2 balance	(80)
miR-150	Control of B cell differentiation, pre-and pro-B cell formation or function	(15)
miR-155	T-and B-cell maturation, the innate immune response	(14, 15)
miR-181a and miR-181b	B-cell differentiation, CD4 + T-cell sensitivity and selection	(14, 17)
miR-182	Promotion of helper T cell-mediated immune responses upon induction by IL-2	(16)
miR-223	Neutrophil proliferation and activation	(14, 15)
miR-451	Regulation of Macrophage migration inhibitory factor (MIF)	(83)
let-7a	Regulate T cell subsets and improve macrophage infiltration	(71)

### Antibacterial and Antiviral Functions

Neonatal immune system development is not mature yet and is vulnerable to pathogenic bacteria, neonatal necrotizing enterocolitis, neonatal sepsis, and other infectious diseases. HBM contains a large number of antibacterial components (89), immune cells, and immune regulatory factors, which can promote the maturation of the immune system and regulate immune tolerance and inflammatory response. The discovery of miRNA in HBM with immunological protection provides a new possibility for the prevention and treatment of neonatal infectious diseases. miRNA-146a in breast milk can regulate the expression of Toll-like receptor 4 (TLR4) through a negative feedback mechanism (17). Yang et al. have found that the miRNA-146a content in neonatal monocytes is significantly higher in 24 h after stimulation with lipopolysaccharide, and has protective effects on infected newborn. It can reduce the incidence of neonatal septic shock and chronic infection. The contents of miRNA-146a and miRNA-223 in the blood of patients with sepsis decreased significantly, which can be used as biomarkers for the diagnosis of sepsis. The decline of miRNA-150 and miRNA-574-5p is related to the severity of sepsis and can be used to evaluate the prognosis of sepsis. The contents of miRNA-146a, miRNA-223, miRNA-150, and miRNA-574-5p in HBM are high. Breastfeeding may play an anti-infective role by increasing these miRNAs level in the blood of infants (97).

HBM exosomes can resist enteroviruses, and exosomes in colostrum can neutralize at least one or several enteroviruses, including poliovirus types 1, 2, and 3, Coxsackievirus A9, B3 and Be, ECHO virus types 6 and 9, and rotavirus. HBM exosomes also can resist respiratory viruses and can resist the infection of influenza virus, parainfluenza virus, adenovirus, and respiratory syncytial virus. In addition to the above viruses, it has been reported that HBM exosomes contain resistance factors to herpes simplex virus and show activity against human immunodeficiency virus, cytomegalovirus, HIV-1, and hepatitis C virus, which is related to their ability to prevent virus-cell adhesion or replication (98, 99). In short, the antiviral characteristics of HBM exosomes can be summarized as follows. firstly, there are specific and non-specific immune components against a variety of viruses in HBM exosomes. Secondly, SIgA plays a major role in these antiviral substances. Thirdly, the titer of antiviral substances in colostrum is high, but it decreases significantly in mature milk.

### HBM Exosomes Have Inherent Antitumor Activity and Targeted Drug Delivery Function

HBM exosome surface expression of MHC molecules, costimulatory molecules can activate *in vivo* antigen-specific T cells to play their immune role. *In vitro* experiments showed that T cells could not be activated directly by exosomes but by the aid of dendritic cells (DC). That is, exosomes secreted by a DC carrying antigen-MHC molecular complex are phagocytosed by another DC, and the DC is stimulated by some signal to mature. The mature DC can be used as a medium to activate specific T cell immune response. In this process, the main role of HBM exosomes is to present the antigen-MHC molecular complex to the DC that is not exposed to the antigen. In this way, the number of DC-carrying antigens increases, and more T cells are activated to cause a strong immune response. In antitumor immunity, HBM exosomes carrying tumor antigen are phagocytosed by DC and use DC as a medium to activate tumor antigen-specific T cells, thus playing an antitumor role (1, 100). With the significant advancements in the research of nucleic acids, proteins, and microbiota, HBM is now known to contain distinct bioactive molecules along with renowned nutritional components, and their therapeutic roles are becoming acknowledged. In particular, HBM exosomes have gained much attention owing to their inherent antitumor activities. These exosomes modulate immune function and suppress the proliferation of various cancer cells *in vitro* and *in vivo*, and their chemotherapeutic function mainly comes from the miRNA with immune-regulatory and tumor-suppressive activities (15).

The fusion of exosomes with liposomes can increase the loading capacity and also retain the targeting capability. Therefore, HBM exosomes have come into focus as “natural carriers” for certain drugs (101). Drug carriers include liposomes, dendrimers, and polymers because they are easy to digest, non-toxic, and stable (102, 103). Aside from all these attributes, HBM exosomes are considered as a promising material to act as drug carriers due to their natural low cytotoxicity, high bioavailability, low immunogenicity, and autophagy in exosomes provides a theoretical approach for the treatment of lysosomal related diseases (104). According to the study of Kim et al., HBM exosomes can serve as oral delivery vehicles for chemotherapeutic agents and siRNAs, and further modification of these exosomes with a tumor-targeting ligand enables the targeted delivery of drugs to the tumor sites (93, 105).

## Discussion

In this paper, the traditional and new methods for exosomes extraction are reviewed, and the advantages and disadvantages, applicable conditions, and application prospects of each method are discussed. In Minimal Information for Studies of Extracellular Vesicles 2018 (MISEV2018), the International Vesicle Association pointed out that ultracentrifugation is the most commonly used exosomes extraction method at present (106). There are also density gradient centrifugation, polymer precipitation method, size exclusion method, immunocapture method, and microfluidic technology to extract exosomes. However, although the extraction technology of exosomes has been greatly improved, achieving high purity, high efficiency and non-injury extraction of exosomes is still the main challenge in the field of exosomes extraction. For example, ultracentrifugation is a mature method for milk exosomes extraction, but this method requires expensive equipment, and centrifugal force will destroy the structure and function of exosomes, and protein contamination also interferes with the extraction of milk exosomes. Although the extraction of milk exosomes by density gradient centrifugation is purer than that by ultracentrifugation, the introduction of extraction media will lead to the contamination of milk exosomes. Polymer precipitation method can efficiently extract milk exosomes, but the precipitation medium will interfere with the purification and yield of milk exosomes. Although size exclusion method is a method for milk exosomes extraction with high purity and little damage, it is only suitable for the extraction of small volume samples due to strict requirements on sample loading volume. Immunocapture method is the most specific method for milk exosomes extraction at present, but its application in large samples is limited by expensive reagents and strict reaction conditions. Microfluidic technology is a new method for milk exosomes extraction, but the stability and repeatability of this method are poor due to the great influence of antibody, filter film, acoustic wave, and electric field intensity.

**FIGURE 3:**
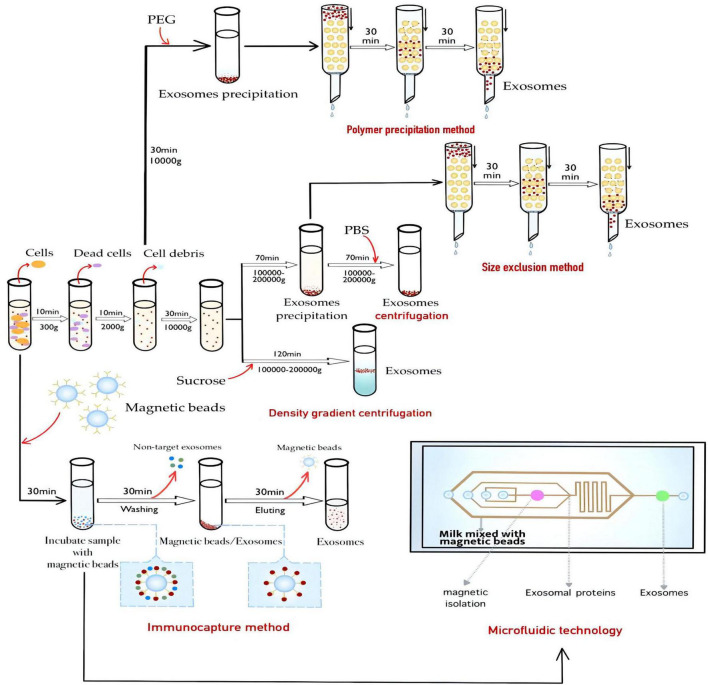
Methods for the extraction of HBM exosomes.

Therefore, the development of efficient and specific milk exosomes isolation and purification methods is still the core of exosomes research and application. The new MISEV guidelines in 2018 suggested the combined use of milk exosomes extraction methods based on recent research progress, to obtain more efficient exosomes than traditional methods (106). At present, it seems that the combination of these methods may be superior to a single milk exosomes extraction method. With the deepening of research and the continuous progress of science and technology, more and more breast milk exosomes extraction technologies and their combinations keep emerging (Figure 3). It is believed that shortly, HBM exosomes extraction technologies will become more mature, and more biological functions of HBM exosomes will be explored. The goal to apply HBM exosomes research in clinical diagnosis and treatment will be realized ultimately.

## Conclusion and Prospects

Nowadays, with the in-depth understanding of the secretion mechanism and function of exosomes, people realize that exosomes, as an important medium of intercellular communication, not only participate in the normal physiological function of organisms but also involve the pathogenesis of some diseases, including tumors, cardiovascular diseases and neurodegenerative diseases. Exosomes-therapy may serve as a new approach to combat aging (107). Exosomes are considered as biomarkers and prognostic factors of diseases. In addition, they can be used as carriers for gene and drugs, which has important clinical diagnostic and therapeutic significance. Based on the currently reported methods for the isolation and extraction of breast milk exosomes, there has not been a complete and mature extraction standard for HBM exosomes (24). Compared with the traditional centrifugal methods, microfluidic technology combined with immunocapture method has the most significant advantage in the extraction of HBM exosomes. Firstly, the extraction sensitivity of these exosomes can be increased by 10-100 times *via* immunocapture method, and its biocompatibility is good. Secondly, specific exosomes adsorbed by magnetic beads can be quickly extracted in a microfluidic device, and the process has been fully automated. It is less affected by interference factors, and the time is short. This combined technology is a promising new exosomes extraction method, which is rapid and efficient. However, this extraction method requires the use of microfluidic devices, which are currently bulky and inconvenient to carry. Therefore, this aspect should be improved in future development, such as simplifying the instrument structure, reducing the size of the instrument to make it easy to carry. The following points need to be paid attention to in the study of HBM exosomes. Firstly, the collection and storage conditions of HBM will have a certain influence on the milk component. Fatty acid failure and temperature changes may have a certain impact on HBM exosomes. Error caused by environmental factors should be avoided in the research process. secondly, the extraction method of HBM exosomes has a certain degree of similarity with that of milk exosomes. However, due to the complexity of the components of HBM, it is necessary to consider pretreatment to remove more fat and protein impurities in HBM. Thirdly, in the extraction of HBM exosomes, a combination of various extraction methods can be considered to the improve efficiency and yield. Last but not least, tissue or cell-specific delivery of the gene vectors by designed HBM exosomes drug carriers enables an advanced version of gene therapy with reduced toxicity or risk.

Compared with other body fluids, HBM exosomes are easier to obtain and more acceptable ethically. At the same time, it has the natural advantage of low immunogenicity, which is favorable when used as drug delivery complex. Further study can also be carried out on the potential biological mechanism of HBM exosomes in disease diagnosis and treatment, so that it can be applied in clinical practice. However, the low sensitivity and specificity of the detection of exosomes could be an obstacle, while tracing the origin of exosomes from different cells through the specific phenotypic characteristics might be challenging as well. In addition, it is difficult to screen exosome markers with high specificity due to the large individual differences of HBM exosomes. And through further differentiation of exosomes subtypes, new breakthroughs to the industrial application of exosomes might be achieved. On the other hand, with the development of the research on exosomes, existing purification technology cannot meet the needs of research. Only by developing more efficient exosome purification technology can we make more in-depth exploration in the field of exosomes.

Future studies are needed to reveal how individual bioactive components within HBM exosomes exert biological functions, including antitumor activity, and to clarify any possible side effects upon HBM exosome treatment. In addition, it would be significant to establish a cost-effective and standardized method to isolate, purify, and manipulate exosomes from HBM to ensure the quality of these exosomes for clinical implementation (54). Combing exosomes with liposomes might create vesicles that can encapsulate large plasmids or miRNA transcripts while retaining the advantages of exosomes such as biocompatibility and targeting capability. Currently, there is no distinct optimal purification technique for the extraction of exosomes with high purity. Existing extraction methods suffer from low yield and the large-scale production of exosomes for clinical studies and post-drug approval is expensive. Hybrid exosome designs are needed for future clinical applications, while possible side effects must be considered. And immunosuppressive effects based on the nature of parental donor cells might be observed due to the heterogeneous components of exosomes. In addition, tumor cells can regulate its microenvironment through exosomes to achieve immune escape (108). Recent studies have found that exosomes secreted by tumor cells such as ovarian cancer can down-regulate the antigen-presenting ability of antigen-presenting cells by carrying immunosuppressive molecules and even induce apoptosis of peripheral blood lymphocytes and DC, which inhibit the antitumor immune response of the body (109). The soluble ligand of NK cell activated receptor (NKG2D) is highly expressed on the surface of exosomes secreted by prostate cancer cells, which can selectively act upon NKG2D on NK cells and CD8 + T cells and downregulate its expression. It is inhibiting NKG2D mediated cytotoxicity and realizes tumor immune escape (110, 111). It can be seen that in the game between the immune system and tumor cells. Exosomes can become the “helper” of the body by enhancing immune surveillance and immune killing on the one hand, while on the other hand, they can also become the “accomplice” of tumor cells by inhibiting antigen presentation and immune cell activity. If such systems are designed their clinical efficacy and safety parameters need to be thoroughly characterized. To functionalize exosomes ligands are attached to the surface through chemical conjugation. These active targeting molecular combinational methods and products need to be investigated. Integrative studies using therapeutic cargo and functional exosomes or hybrid exosome mimetics are drawing attention. Exosomes that carry caspase-3 may also inhibit cell death by apoptosis or enhance tumor cell survival by preventing chemotherapeutics drug accumulation. One of the approaches is to design artificial exosomes or exosome mimetics that can overcome potential disadvantages such as unwanted immunosuppressive (112). To summarize, with the advancement and optimization of gene-editing tools, the continuous development of exosome-based carrier systems will power the targeted gene therapies as accessible treatments for difficult and complicated diseases in the future, especially neonatal diseases, through both *in vitro* and *in vivo* routes (112, 113).

## Author Contributions

XL: conceptualization, methodology, writing—original draft preparation, investigation, and formal analysis. LS: investigation and writing. XZ: writing—review. QC: data collection and organization. YW, ZS, TZ, LW, and YX: review and editing. XF: writing—review and editing. XY: resources, writing—review and editing, supervision, project administration, and funding acquisition. All authors contributed to the article and approved the submitted version.

## Conflict of Interest

The authors declare that the research was conducted in the absence of any commercial or financial relationships that could be construed as a potential conflict of interest.

## Publisher’s Note

All claims expressed in this article are solely those of the authors and do not necessarily represent those of their affiliated organizations, or those of the publisher, the editors and the reviewers. Any product that may be evaluated in this article, or claim that may be made by its manufacturer, is not guaranteed or endorsed by the publisher.
